# Efficacy, Safety, and Tolerability of Inclisiran in Patients With Homozygous Familial Hypercholesterolemia: Results From the ORION-5 Randomized Clinical Trial

**DOI:** 10.1161/CIRCULATIONAHA.122.063460

**Published:** 2023-10-18

**Authors:** Frederick Raal, Ronen Durst, Ran Bi, Zsolt Talloczy, Pierre Maheux, Anastasia Lesogor, John J.P. Kastelein

**Affiliations:** 1 Faculty of Health Sciences, University of the Witwatersrand, Johannesburg, South Africa (F.R.).; 2 Cardiology Department, Hadassah-Hebrew University Medical Centre, Jerusalem, Israel (R.D.).; 3 Novartis Pharmaceuticals Corporation, East Hannover, NJ (R.B., Z.T.).; 4 Novartis Pharmaceuticals Corporation, Basel, Switzerland (P.M., A.L.).; 5 Department of Vascular Medicine, Academic Medical Center, University of Amsterdam, Netherlands (J.J.P.K.).

**Keywords:** cholesterol, homozygous familial hypercholesterolemia, lipoproteins, LDL, therapeutics

## Abstract

**BACKGROUND:**

Homozygous familial hypercholesterolemia is a genetic disease characterized by extremely high levels of low-density lipoprotein cholesterol (LDL-C) and a high risk of premature cardiovascular events. The proof-of-concept study ORION-2 (A Study of Inclisiran in Participants With Homozygous Familial Hypercholesterolemia) showed that inclisiran, a small interfering RNA that prevents production of the hepatic PCSK9 protein (proprotein convertase subtilisin/kexin type 9), could lead to durable reductions in LDL-C levels when added to statins and ezetimibe in patients with homozygous familial hypercholesterolemia.

**METHODS:**

ORION-5 was a phase 3, 2-part, multicenter study in 56 patients with homozygous familial hypercholesterolemia and elevated LDL-C levels despite maximum tolerated doses of LDL-C–lowering therapies with or without lipoprotein apheresis. Patients eligible for part 1 (double-blind, 6 months) were randomized 2:1 to receive either 300 mg of inclisiran sodium (equivalent to 284 mg of inclisiran) or placebo. Placebo-treated patients from part 1 were transitioned to inclisiran in part 2 (open-label, 18 months). The primary end point was the percentage change in LDL-C levels from baseline to day 150.

**RESULTS:**

The mean age of the patients was 42.7 years, and 60.7% were women. The mean baseline LDL-C levels were 294.0 mg/dL and 356.7 mg/dL in the inclisiran and placebo groups, respectively. The placebo-corrected percentage change in LDL-C level from baseline to day 150 was −1.68% (95% CI, −29.19% to 25.83%; *P*=0.90), and the difference was not statistically significant between the inclisiran and placebo groups. The placebo-corrected percentage change in PCSK9 levels from baseline to day 150 was −60.6% with inclisiran treatment (*P*<0.0001); this was sustained throughout the study, confirming the effect of inclisiran on its biological target of PCSK9. No statistically significant differences between the inclisiran and placebo groups were observed in the levels of other lipids and lipoproteins (apolipoprotein B, total cholesterol, and non−high-density lipoprotein cholesterol). Adverse events and serious adverse events did not differ between the inclisiran and placebo groups throughout the study.

**CONCLUSIONS:**

Inclisiran treatment did not reduce LDL-C levels in patients with homozygous familial hypercholesterolemia despite substantial lowering of PCSK9 levels. Inclisiran was well-tolerated, and the safety findings were consistent with previously reported studies and the overall safety profile.

**REGISTRATION:**

URL: https://www.clinicaltrials.gov; Unique identifier: NCT03851705.

**Clinical Perspective**

**What Is New?**

- This 24-month study evaluated the efficacy, safety, and tolerability of inclisiran in patients with homozygous familial hypercholesterolemia and elevated low-density lipoprotein cholesterol (LDL-C) levels despite maximum tolerated doses of LDL-C–lowering therapies with or without lipoprotein apheresis.
- There was no statistically significant difference in LDL-C reduction from baseline to day 150 between the placebo and the inclisiran group despite a reduction in PCSK9 (proprotein convertase subtilisin/kexin type 9) levels of 60% that was sustained throughout the study.

**What Are the Clinical Implications?**

- Although this study did not show a statistically significant reduction in LDL-C level in patients with homozygous familial hypercholesterolemia, inclisiran demonstrated a sustained reduction in PCSK9 levels and was well-tolerated, with a safety profile consistent with previous studies.
- Despite the lack of significant LDL-C reduction with inclisiran, the observations in the subsets with some residual *LDLR* function may warrant further investigation.

**Editorial, see p 363**

Homozygous familial hypercholesterolemia (HoFH) is a rare genetic disease that affects ≈1 in 300 000 people worldwide.^1^ HoFH is characterized by elevated low-density lipoprotein cholesterol (LDL-C) levels from birth and is associated with atherosclerotic cardiovascular disease (ASCVD) and sudden death if untreated.^2,3^ More than 90% of sequence variations in HoFH occur in the *LDLR* gene; sequence variations in other genes, such as *APOB*, *PCSK9*, and *LDLRAP1*, have also been reported.^2,3^ The sequence variations that cause a virtually complete absence of *LDLR* activity (null homozygotes with 2 null alleles) result in higher LDL-C levels than alterations that only partially reduce *LDLR* activity by either 2 non-null alleles (non-null homozygotes) or 1 null and 1 non-null allele.^4,5^

Lowering LDL-C levels in patients with HoFH reduces the incidence of cardiovascular events^6^; however, conventional LDL-C–lowering therapies and combinations of these drugs with or without lipoprotein apheresis do not adequately lower LDL-C levels in patients with HoFH. Thus, HoFH is difficult to treat and patients require novel therapeutic options.^7,8^ Statins and ezetimibe reduce LDL-C levels only modestly in patients with HoFH.^3^ As a result, the majority of patients with HoFH do not reach guideline-recommended LDL-C goals and need better therapeutic options with greater LDL-C–lowering effects.^9^

The phase 2 ORION-2 (URL: https://www.clinicaltrials.gov; Unique identifier: NCT02963311) and phase 3 ORION-9 (URL: https://www.clinicaltrials.gov; Unique identifier: NCT03397121) showed that inclisiran, a novel, double-stranded, small interfering RNA–based therapy that prevents the production of the hepatic PCSK9 protein (proprotein convertase subtilisin/kexin type 9), leads to robust and sustained reductions in LDL-C and PCSK9 levels when added to statins and ezetimibe in patients with HoFH and heterozygous familial hypercholesterolemia, respectively.^10–12^ Here, we report the efficacy, safety, and tolerability of inclisiran in patients with HoFH from the 2-part phase 3 ORION-5 trial.

## METHODS

The data that support the findings of this study are available from the corresponding author upon reasonable request.

### Study Design and Treatment

ORION-5 (URL: https://www.clinicaltrials.gov; Unique identifier: NCT03851705) was a multicenter, randomized, double-blind/open-label phase 3 trial conducted in 13 centers across 8 countries (Hong Kong, Israel, Russia, Serbia, South Africa, Taiwan, Turkey, and Ukraine). The objective of the study was to evaluate the efficacy, safety, and tolerability of inclisiran treatment in patients with HoFH and elevated LDL-C levels despite the maximum tolerated doses of standard LDL-C–lowering therapies.

The study had 2 sequential parts. Part 1 consisted of a 6-month double-blind placebo-controlled phase in which eligible patients were randomized 2:1 to receive either 300 mg of inclisiran sodium (equivalent to 284 mg of inclisiran) subcutaneously or placebo at day 1 and day 90. Part 2 consisted of an 18-month open-label, single-arm phase in which patients already receiving inclisiran continued treatment, and placebo-treated patients from part 1 were transitioned to inclisiran and received their first dose of inclisiran subcutaneously on day 180. All patients in part 2 received subsequent doses of inclisiran on days 270, 450, and 630, with an end-of-study visit on day 720. The study design of ORION-5 is presented in Figure S1.

### Study Population

The study included participants aged ≥18 years with genetic confirmation or clinical diagnosis of HoFH on the basis of a history of an untreated LDL-C concentration >500 mg/dL (13 mmol/L) together with either xanthoma before 10 years of age or evidence of heterozygous familial hypercholesterolemia in both parents. Patients were eligible for inclusion if they had a fasting LDL-C level ≥130 mg/dL (≥3.4 mmol/L) despite receiving a maximally tolerated dose of statin therapy with or without ezetimibe. Patients receiving anti-PCSK9 therapies within 90 days of screening were excluded. Details on the inclusion and exclusion criteria are provided in the Appendix in the Supplemental Material.

All patients were instructed to follow a National Cholesterol Education Program Adult Treatment Panel III (or comparable) diet and required to maintain their current LDL-C–lowering drug therapy for the duration of the study.

Patients with a documented regimen of LDL or plasma apheresis were allowed to continue the regimen during the study with similar frequency and timing as baseline. When apheresis was performed on a study visit (dosing or non-dosing visit), blood samples for the measurement of LDL-C and other lipid levels were collected and laboratory assessments were undertaken before the apheresis. When apheresis was performed on a dosing visit, inclisiran was administered just after the completion of apheresis. No apheresis was performed within 72 hours after dosing of inclisiran. The subsequent study visits were planned to occur at least 2 weeks after apheresis to ensure LDL-C levels measured during a study visit were not confounded by apheresis.

### Genotyping

Genotyping was performed by a central laboratory or was available from the medical records of the study population. On the basis of this assessment, patients were categorized as homozygous *LDLR* (identical *LDLR* pathogenic variants), compound heterozygous *LDLR* (different *LDLR* pathogenic variants), double null for *LDLR* (null/null patients who have minimal residual *LDLR* activity as both variants are nonfunctional), or other genetic types. *LDLR* activity assumptions were derived from the Global Variome shared LOVD and ClinVar databases.^13,14^ A null qualifier was given if the reported *LDLR* activity was ≤15%.

### Ethical Considerations

ORION-5 was conducted in accordance with the International Council for Harmonisation of Technical Requirements for Pharmaceuticals for Human Use E6 guideline for Good Clinical Practice, which has its origin from the Declaration of Helsinki. The study protocol and all amendments were approved by the independent ethics committee or institutional review board of all participating centers. All patients provided written informed consent.

### Data Sharing Statement

Novartis is committed to sharing access to patient-level data and supporting clinical documents from eligible studies with qualified external researchers. These requests are reviewed and approved by an independent review panel on the basis of scientific merit. All data provided are anonymized to respect the privacy of patients who have participated in the trial in line with applicable laws and regulations. The availability of these trial data is according to the criteria and process described at www.clinicalstudydatarequest.com.

### Efficacy End Points

The primary end point was the percentage change in LDL-C level from baseline to day 150 (part 1 of the study). Key secondary end points were the absolute change in LDL-C level from baseline to day 150 and the percentage change in apoB (apolipoprotein B100), non–high-density lipoprotein cholesterol (non-HDL-C), and total cholesterol levels from baseline to day 150. Other secondary end points were the absolute and percentage changes in LDL-C, PCSK9, apoB, non–HDL-C, lipoprotein(a), and total cholesterol levels from baseline to day 720.

An exploratory end point evaluated the response of LDL-C level reduction by underlying causal sequence variations of HoFH at day 150. A post hoc analysis was conducted to evaluate the placebo-corrected percentage change in LDL-C level from baseline, excluding patients with apheresis and double-null alleles for *LDLR* (null/null).

### Safety End Points

The safety and tolerability profile of inclisiran was measured by adverse events (AEs), serious AEs, and clinical laboratory values. In addition, an anti-inclisiran antibody analysis was performed. AEs were assessed from baseline to each assessment time up to day 720. Data captured during part 1 (up to day 180) were presented by the part 1 treatment group (inclisiran or placebo), whereas data captured during part 2 (after day 180 up to day 720) were presented by the part 2 treatment group (inclisiran–inclisiran or placebo–inclisiran).

### Statistical Analysis

#### Sample Size Calculation

It was calculated that a sample size of at least 45 patients (randomized 2:1 to inclisiran:placebo), with at least 30 patients in the inclisiran arm, would provide >80% power to detect a 20% reduction (assuming an SD of 20% in each arm) in placebo-corrected LDL-C levels from baseline to day 150 in the inclisiran group compared with the placebo group at a 1-sided significance level of 0.025 based on a 2-sample *t* test. Demographic and baseline characteristics of patients were summarized descriptively in the intent-to-treat population comprising all randomized patients.

#### Missing Data Imputation

Missing values in part 1 were imputed for the primary and key secondary end points using a prespecified multiple imputation (a total of 100 imputed data sets) washout model. The washout model can be thought of as a modified control-based pattern–mixture model. This was used to explore the possibility of data missing not at random for patients who discontinued the study early. For patients who discontinued the study early in the inclisiran group, their missing values for day 150 were imputed under the assumption that their outcome would be similar to those in the placebo group with similar background characteristics. For patients in the placebo group, their missing values over all visits after early termination were imputed based on the missing at random assumption. Multiple imputation was used to account for uncertainty in the imputation process and results from the imputed data sets were combined using the Rubin method.^15^

#### Efficacy Analysis

The primary analysis was conducted on the intent-to-treat population and based on an ANCOVA model on the percentage change in LDL-C level from baseline to day 150 on each multiply imputed data set (100 total). The model included the part 1 treatment group as a fixed effect and the baseline LDL-C level as a covariate. Treatment effects from these 100 ANCOVA analyses were combined using the Rubin method.^15^ The difference in the least squares mean between treatment groups, corresponding 2-sided 95% CIs, and *P* values based on *t* test for testing equal mean percentage change from baseline to day 150 in LDL-C level are provided.

The key secondary end points were analyzed from a similar ANCOVA model using the same multiply-imputed washout model as the primary efficacy end point. For the other secondary end points, descriptive and graphical summaries by treatment group were presented. The mixed-effects model for repeated measures was performed to analyze other secondary end points, such as the absolute or percentage change from baseline over time up to day 180 for LDL-C, PCSK9, and other lipid levels. The model included fixed effects of the part 1 treatment group, visit (day 90, day 150, and day 180), baseline value, and the interaction between treatment and visit. The restricted maximum likelihood estimation approach was used with the covariance structure set as “unstructured.” The least squares means were calculated for each treatment at each visit, and linear combinations of the estimated least squares mean were used to form *t* tests to assess the differences between treatment groups.

The analysis of the exploratory end point was similar to that of the primary end point. The post hoc analysis was conducted similar to that of the other secondary end points.

All end points were tested using a 2-sided significance level of 0.05. The *P* values reported throughout the article are 2-sided for testing the equal mean percentage change (or absolute change) between treatment groups.

#### Safety Analysis

Safety variables were summarized descriptively by parts and treatment groups. Safety was analyzed in the safety population comprising all patients who received ≥1 dose of the study drug using the standard Medical Dictionary for Regulatory Activities nomenclature.

All analyses were performed with SAS software (version 9.4).

## RESULTS

### Baseline Demographic Characteristics

Overall, 56 patients were enrolled, of whom 37 were randomized to the inclisiran group and 19 to the placebo group. A total of 53 patients (94.6%) completed part 1, 47 of whom (88.7%) also completed part 2. The reasons for discontinuation were death, withdrawal of consent, or “other” (Figure S2).

Overall, the mean (SD) age of patients was 42.7 (12.9) years, and 60.7% were women. A total of 67.9% of patients had established ASCVD, and 32.1% had an ASCVD risk equivalent (Table 1). At baseline, all patients received high-intensity statin therapy, and the mean (SD) LDL-C levels were 294.0 (136.3) mg/dL and 356.7 (122.4) mg/dL in the inclisiran and placebo groups, respectively. The proportion of patients with a family history of premature cardiovascular disease in a first-degree relative was lower in the inclisiran group (54.1%) compared with the placebo group (73.7%). Baseline characteristics were balanced between the inclisiran and placebo groups, except for double null alleles for *LDLR* (inclisiran, n=10 [27.0%]; placebo, n=3 [15.8%]; Table S1).

**Table 1.**
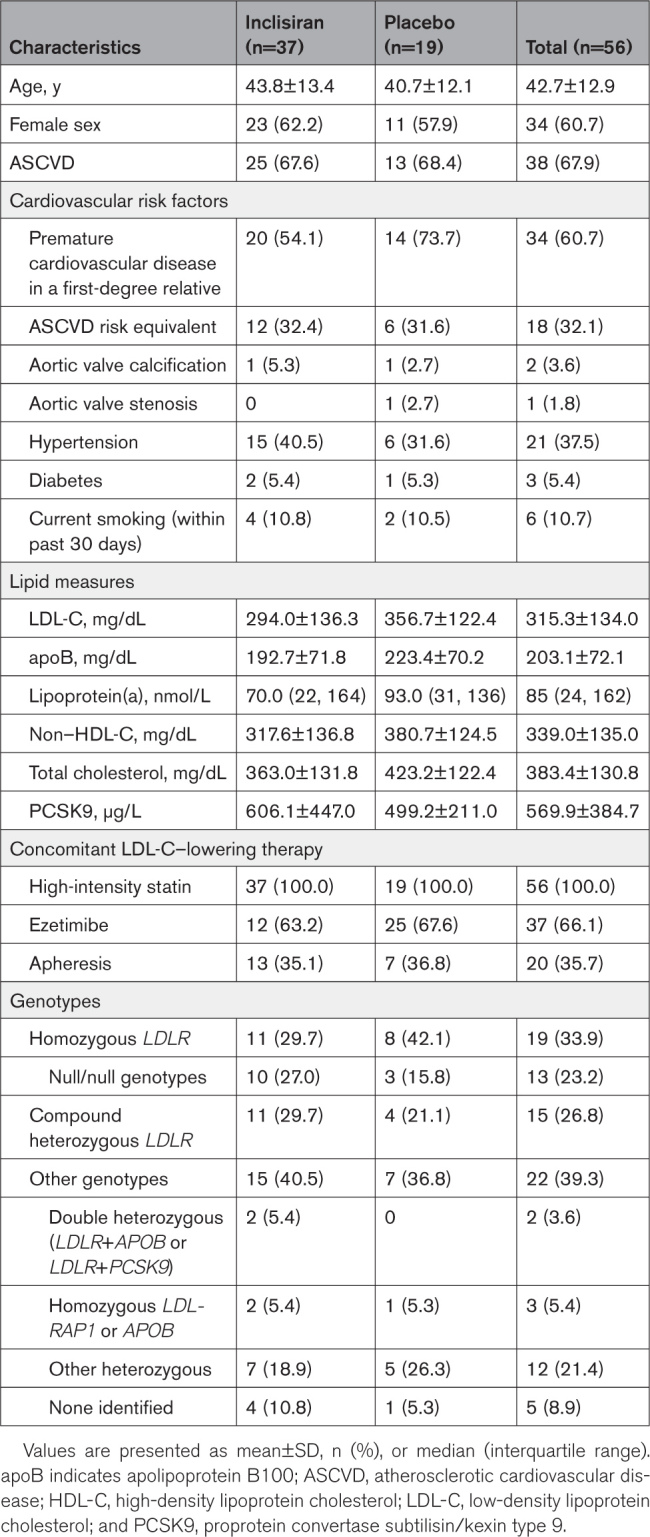
Demographic and Baseline Characteristics (Intent-to-Treat Population)

### Primary End Point

**Figure 1.**
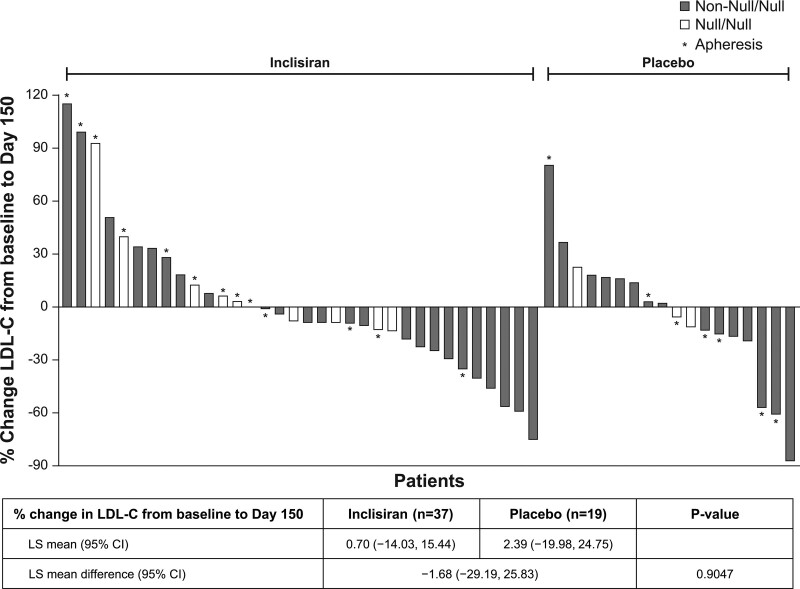
**Waterfall plot of percentage change in LDL-C level from baseline to day 150 for patients with LDL-C data (intent-to-treat population).** Reflexive low-density lipoprotein cholesterol (LDL-C) was used for the analysis. The total number of observed cases was 52. A multiple imputation washout model was used for missing data imputation with 100 total imputed data sets. ANCOVA on each of the 100 data sets was performed by including the fixed effect for double-blind treatment and baseline LDL-C as a covariate, assuming unequal variances between treatment groups. Treatment effects from the 100 analyses were combined using the Rubin method. LS indicates least squares.

The placebo-corrected percentage change in LDL-C level from baseline to day 150 for part 1 was −1.68% (*P*=0.90). There was no statistically significant difference in the reduction in LDL-C level from baseline to day 150 between the placebo and the inclisiran groups (Figure 1). The variability in the percentage change in LDL-C level from baseline to day 150 is presented in a waterfall plot (Figure 1). At day 150, 58.8% of patients (n=20) in the inclisiran group had a reduction in LDL-C level from baseline, compared with 50.0% (n=9) in the placebo group. However, no statistically significant difference was observed between the treatment groups.

### Key Secondary End Points

The placebo-corrected absolute change in LDL-C level from baseline to day 150 was 6.47 mg/dL, which was not statistically significant (*P*=0.87). The placebo-corrected percentage changes from baseline to day 150 for apoB (−4.3%; *P*=0.68), non–HDL-C (−2.1%; *P*=0.87), and total cholesterol (−0.8%; *P*=0.94) were also not statistically significant.

### Other Secondary End Points

The placebo-corrected absolute and percentage changes from baseline by visit for LDL-C, apoB, non–HDL-C, lipoprotein(a), and total cholesterol levels were not statistically significant at any time point.

The reductions in PCSK9 levels from baseline to days 90, 150, and 180 were consistently greater in the inclisiran group than in the placebo group and were statistically significant. The placebo-corrected absolute change in PCSK9 level from baseline to day 180 ranged between −304.4 µg/L (95% CI, −408.0 µg/L to −200.8 µg/L) and −390.4 µg/L (95% CI, −504.5 µg/L to −276.3 µg/L; *P*<0.0001 for all time points), and the placebo-corrected percentage change in PCSK9 level from baseline to day 180 ranged between −60.6% (95% CI, −83.5% to −37.8%) and −92.3% (95% CI, −120.4% to −64.2%; *P*<0.0001 for all time points). In part 1, inclisiran treatment consistently reduced PCSK9 level from baseline at day 90 up to day 180 by ≈55%, and showed a greater and statistically significant PCSK9 reduction compared with the placebo group (Figure S3). Similar reductions in PCSK9 levels (≈55%) were observed in part 2 for both the inclisiran–inclisiran and placebo–inclisiran groups.

### Exploratory Subgroup

**Figure 2.**
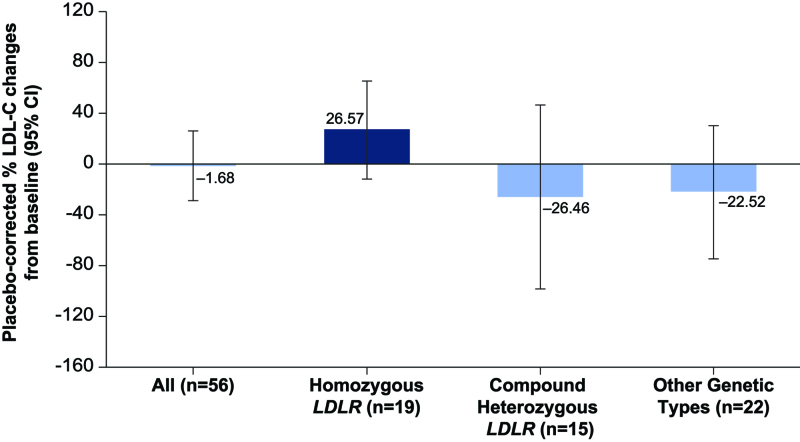
**Placebo-corrected percentage change in LDL-C level from baseline to day 150 by genotype subgroup.** Subgroups included homozygous *LDLR* (inclisiran, n=11; placebo, n=8; *P*=0.18), compound heterozygous (inclisiran, n=11; placebo, n=4; *P*=0.47), and other genetic types (inclisiran, n=15; placebo, n=7; *P*=0.40).

The distribution of genotypes showed that 19 patients had homozygous *LDLR*, 15 patients had compound heterozygous *LDLR*, and 22 patients had other genetic type sequence variations. In a subgroup analysis by genotype that evaluated the reduction in LDL-C level from baseline to day 150 by underlying causal sequence variations of HoFH, the placebo-corrected percentage reduction in LDL-C level from baseline to day 150 was numerically greater in the compound heterozygous *LDLR* group (−26.5%; n=15; *P*=0.47) than the other genetic types group (−22.5%; n=22; *P*=0.40), but LDL-C level increased in the homozygous *LDLR* group (+26.6%; n=19; *P*=0.18). These differences were not statistically significant (Figure 2).

Post hoc analysis to evaluate the placebo-corrected percentage changes in LDL-C level from baseline excluding patients with apheresis and the null/null genotype (n=30), 2 factors that can affect the treatment effect on LDL-C levels besides the study treatment received, showed greater reduction in LDL-C level compared with all patients. The placebo-corrected least squares mean percentage change in LDL-C level from baseline at each visit up to day 180, excluding those patients with apheresis and the null/null genotype, ranged between −12.9% and −30.0%, whereas it was between +4.6% and −12.1% for all patients.

### Safety Evaluation

A summary of the treatment-emergent AEs and the treatment-emergent serious AEs in part 1 and in part 2 is shown in Table 2.

**Table 2.**
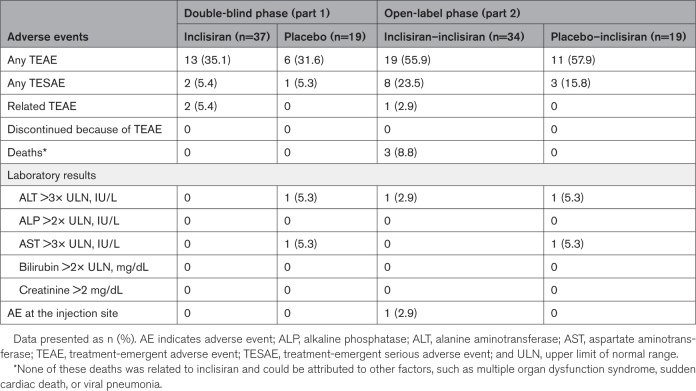
Treatment-Emergent Adverse Events (Safety Population)

The most commonly reported treatment-emergent AEs did not differ between treatment groups in either part of the study. In part 1, the most common treatment-emergent AEs were viral respiratory tract infection (n=2 [5.4%] inclisiran; n=2 [10.5%] placebo), diarrhea, and pyrexia (n=2 [5.4% inclisiran]; 0 placebo); in part 2, they were coronavirus infection (n=3 [8.8%] inclisiran–inclisiran; n=1 [5.3%] placebo–inclisiran) and an increased international normalized ratio (n=2 [5.9%] inclisiran–inclisiran; n=1 [5.3%] placebo–inclisiran). During the entire study, only one patient (2.9%) had an AE at the injection site (erythema); this was in the inclisiran–inclisiran group during part 2, and the AE was mild in severity, not persistent, and resolved rapidly.

Although the AEs and serious AEs were based on a small number of events reported in a small data pool, the incidences of these did not differ between the inclisiran and placebo groups throughout the entire study or between the parts of the study. Three deaths were reported during the study, all during part 2. None of these deaths were related to inclisiran treatment, and all were attributed by the investigator to other factors (multiple organ dysfunction syndrome, sudden cardiac death, and viral pneumonia). No anti-inclisiran antibodies were detected in any of the samples during the study.

## DISCUSSION

ORION-5 did not meet its primary objective of detecting a statistically significant difference in the percentage change in LDL-C levels from baseline to day 150 between the inclisiran and placebo groups. Placebo-corrected changes in PCSK9 levels from baseline to day 150 were observed with inclisiran treatment and were sustained throughout the study, confirming the effect of inclisiran on its biological target of PCSK9. No statistically significant changes were observed in the levels of other lipids and lipoproteins (apoB, non–HDL-C, lipoprotein[a], and total cholesterol) between the inclisiran and placebo groups.

In the ORION-2 proof-of-concept study, 3 of the 4 patients with HoFH showed a substantial reduction in LDL-C levels that was maintained for 180 days.^10^ In the phase 3 ORION-9 trial, inclisiran treatment in patients with heterozygous familial hypercholesterolemia resulted in a placebo-corrected reduction of 47.9% in LDL-C and 78.3% in PCSK9 levels from baseline to day 510.^12^ However, despite engagement and reduction in its biological target of PCSK9 levels, the current study could achieve a placebo-corrected reduction of only 1.68% in LDL-C and 60.6% in PCSK9 levels from baseline to day 150 in patients with HoFH with a similar dose of inclisiran. Although these differences in the treatment effect can be attributed to the diverse genotype sequence variations and the higher production rate of PCSK9 in patients with HoFH than with heterozygous familial hypercholesterolemia, it is likely that inclisiran requires sufficient residual *LDLR* function to be effective in patients with HoFH.

Patients with HoFH often show high genetic variability, and the response to LDL-C–lowering therapies is affected by sequence variations mainly related to the *LDLR* gene, with the worst responses observed in patients with a homozygous *LDLR* genotype, particularly those with null/null *LDLR* sequence variations.^16–18^ Furthermore, *LDLR* expression varies considerably in patients with HoFH, even in those with identical *LDLR* sequence variations, and marked interindividual variability in response to LDL-C–lowering therapies has been observed in these patients treated with anti-PCSK9 monoclonal antibodies.^17,19–22^ This variability in the response to LDL-C–lowering therapies remains unexplained; however, residual *LDLR* expression in patients with HoFH is suggested to be an important contributor.^19,23^ In this study, there was substantial variability in the LDL-C–lowering response to inclisiran treatment, possibly because of the different genotype subgroups with varying treatment effects; the reduction in LDL-C level was of greater magnitude in patients with higher residual *LDLR* function. However, the small study size limited any further subgroup analysis, and these results need to be explored further in a large group of patients before inclisiran can be considered as a treatment option for patients with HoFH with high residual *LDLR* function.

A small study population with a heterogeneous set of genetic variants might have affected the treatment responses to inclisiran in the current study, leading to the study not meeting its primary end point and several secondary end points. There were 13 null/null patients with a homozygous or compound heterozygous *LDLR* sequence variation, but a disproportionate number of null/null *LDLR* patients were randomized to the inclisiran group (n=10 [27.0%]) than to the placebo group (n=3 [15.8%]), and this might have confounded the results. These findings are consistent with other studies that have suggested that variant status alone can have a significant effect on LDL-C–lowering treatment response to LDL-C–lowering therapies.^22,24^

In the real world, suboptimal adherence and persistence to daily LDL-C–lowering therapy is the most likely reason for not achieving the guideline-recommended LDL-C goals in many patients.^25,26^ Patients with previous use (n=17 of 54 [31.5%]) and treatment failure of anti-PCSK9 therapies (n=5) were included in the current study, and some of these nonresponders to previous anti-PCSK9 therapies might also have been nonresponders to inclisiran.

Lipoprotein apheresis is often used as an adjunct therapy in patients with HoFH who do not achieve sufficient lowering of LDL-C level despite optimum LDL-C–lowering therapy.^7,8^ However, apheresis can be a strong confounder in the evaluation of the treatment effect of other therapies. A similar proportion of patients in the inclisiran (35.1%) and placebo (36.8%) groups had apheresis in the current study. However, the frequency of apheresis and the methods used for LDL-C measurement in patients who had apheresis can affect the measurement of percentage changes in LDL-C levels. Although the percentage change in LDL-C levels during the study was measured after a gap of ≥2 weeks after apheresis, it is plausible that the effect of apheresis administered to patients before randomization, close to the time of the baseline measurement for LDL-C levels, could have lowered their baseline LDL-C values and thereby hindered the treatment difference between the inclisiran and placebo groups. The baseline LDL-C levels in the inclisiran group (294.0 mg/dL) were far lower than those in the placebo group (356.7 mg/dL), and this also might have affected the comparison of the treatment difference between the inclisiran and placebo groups.

HoFH is difficult to treat, and patients often require treatment with multiple therapies.^27^ Although PCSK9-directed therapies remain a valuable treatment strategy and should be continued in treatment responders, nonresponders should be switched to alternative treatment therapies that work independently of *LDLR* function for further control of LDL-C.

### Conclusions

In this study, although inclisiran treatment did not result in a statistically significant reduction in LDL-C level compared with placebo, the sustained reduction in PCSK9 levels demonstrates target engagement with inclisiran even in patients with HoFH. Inclisiran was well-tolerated, and the safety findings are consistent with previously reported studies and the overall safety profile. No new safety signals were identified during the study.

## ARTICLE INFORMATION

### Acknowledgments

The authors thank Ganesh Pedgaonkar, PhD, Vennila Dharman, MBBS, and Ritika Paul, MSc, MPhil, of Novartis Healthcare Pvt Ltd, for providing medical writing support in accordance with Good Publication Practice 2022 guidelines (Good Publication Practice Guidelines for Company-Sponsored Biomedical Research; 2022 Update; *Annals of Internal Medicine*; acpjournals.org), and Klaus Molle, PhD, of Novartis Pharma AG, for critical review of the manuscript and editorial guidance.

### Sources of Funding

Novartis Pharma AG initiated and provided funding for this study and provided medical writing and editorial support in the development of the manuscript. The funder was also involved in study design and data collection, analysis, and interpretation, as well as review and feedback on the manuscript. The authors, who include both academic investigators and employees of the funder, had full editorial control of the manuscript and provided final approval of all content. All authors had access to the data and contributed to the review of and revisions to manuscript drafts. All authors vouch for the accuracy and completeness of the data and for the fidelity of the trial to the protocol.

### Disclosures

Dr Raal received consulting fees and honoraria from Amgen, Novartis Pharmaceuticals, Regeneron Pharmaceuticals, and LIB Therapeutics. Dr Durst received consulting fees and honoraria from Novartis and Sanofi, lecturing fees from Novartis, and honoraria from Medison. Drs Bi, Talloczy, Maheux, and Lesogor are full-time employees of Novartis and have Novartis stocks or stock options. Dr Kastelein received consulting fees from 89Bio, Esperion Therapeutics, Madrigal, CiVi Therapeutics, North Sea Therapeutics, CSL-Behring, Novartis, Inversago, Draupnir, CinCor, and Scribe Therapeutics; is a board member of North Sea and Staten Biotech; is Chief Scientific Officer of New Amsterdam Pharma; is acting Chief Medical Officer of Staten Biotech; and holds stock and stock options in New Amsterdam.

### Supplemental Material

Appendix

Table S1

Figures S1–S3

## Supplementary Material
