# Supplementary material for: Efficacy, Safety, and Tolerability of Inclisiran in Patients With Homozygous Familial Hypercholesterolemia: Results From the ORION-5 Randomized Clinical Trial
Source: Circulation. 2023 Oct 18;149(5):354–62. doi: 10.1161/CIRCULATIONAHA.122.063460 (PMC10815002; doi:10.1161/CIRCULATIONAHA.122.063460)
Supplement: Supplementary file 1 [file cir-149-354-s001.pdf]

SUPPLEMENTAL MATERIAL

Supplementary Appendix

Inclusion criteria

1. Adult patients (aged ≥18 years) with confirmed diagnosis of HoFH by genetic testing or a clinical diagnosis based on a history of an untreated LDL-C concentration >500 mg/dL (13 mmol/L) together with either xanthoma before 10 years of age or evidence of HeFH in both parents

2. Stable on a low-fat diet

3. Fasting central laboratory LDL-C concentration ≥130 mg/dL (3.4 mmol/L)

4. Triglyceride concentration <400 mg/dL (4.5 mmol/L)

5. Patients on statins were to be receiving a maximally tolerated dose, defined as the maximum dose of statin that can be taken on a regular basis without intolerable AEs. Patients not receiving statins were required to have documented evidence of intolerance to at least two different statins.

6. Patients on LDL-C–lowering therapies (such as a statin and/or ezetimibe) were to be on a stable dose for ≥30 days before screening with no planned medication or dose change during study participation

7. No current or planned renal dialysis or renal transplantation

8. Patients on a documented regimen of LDL-C or plasma apheresis were allowed to continue the apheresis during the study, if needed

Exclusion criteria

1. Any uncontrolled or serious disease, or any medical or surgical condition, that could have either interfered with participation in the clinical study, and/or put the patient at significant risk (according to the investigator's judgment) if he/she participated in the clinical study

2. Use of mipomersen or lomitapide therapy within 5 months of screening

3. Any underlying known disease or surgical, physical, or medical condition that, in the opinion of the investigator (or delegate) might have interfered with interpretation of the clinical study results

4. New York Heart Association (NYHA) class IV heart failure or last known left ventricular ejection fraction <25%

5. Major adverse cardiovascular event within 3 months prior to randomization

6. Uncontrolled severe hypertension: systolic blood pressure >180 mmHg or diastolic blood pressure >110 mmHg prior to randomization despite anti-hypertensive therapy

7. Active liver disease defined as any known current infectious, neoplastic, or metabolic pathology of the liver or unexplained elevations >3x the upper limit of normal (ULN) in alanine aminotransferase, aspartate aminotransferase, or >2x ULN total bilirubin at screening confirmed by a repeat abnormal measurement at least 1 week apart

8. Severe concomitant non-cardiovascular disease that carries the risk of reducing life expectancy to less than the duration of the trial

9. History of malignancy that required surgery (excluding local and wide-local excision), radiation therapy and/or systemic therapy during the 3 years prior to randomization

10. Pregnant or nursing females, or those who were of childbearing potential and unwilling to use at least one method of highly effective contraception (failure rate less than 1% per year) (e.g. combined oral contraceptives, barrier methods, approved contraceptive implant, long-term injectable contraception, or intrauterine device) for the entire duration of the study. Exemptions from this criterion:

   a. Women >2 years postmenopausal (defined as ≥1 year since last menstrual period) and >55 years of age

    b. Postmenopausal women (as defined above) and <55 years of age with a negative pregnancy test within 24 hours of enrollment
    c. Women who were surgically sterilized ≥3 months prior to enrollment

11. Known history of alcohol and/or drug abuse within the last 5 years
12. Treatment with other investigational products or devices within 30 days or 5 half-lives of the screening visit, whichever was longer
13. Planned cardiac surgery or revascularization during the course of the study
14. Treatment (within 90 days of screening) with anti- PCSK9 therapies
15. Previous participation in the study
16. Hypersensitivity to any of the ingredients of inclisiran

Table

Table S1: Mutations in LDLR and assigned functional status

Age	Sex	Treatment	Genotype	Gene affected	Mutation type	LDLR function
25	F	Inclisiran	Homozygous LDLR	*LDLR*	c.681C>G (plus single allele PCSK9 c.599T>C)	Null/Null
38	M	Inclisiran	Homozygous LDLR	*LDLR*	c.2054C>T (plus a single allele APOB c.1272G>T)	Null/Null
50	M	Placebo	Homozygous LDLR	*LDLR*	c.664T>C	Non-Null/Null
53	F	Inclisiran	Homozygous LDLR	*LDLR*	c.1324T>C	Non-Null/Null
29	F	Placebo	Homozygous LDLR	*LDLR*	c.1823C>T	Non-Null/Null
37	M	Placebo	Homozygous LDLR	*LDLR*	c.1567G>A	Non-Null/Null
32	F	Inclisiran	Homozygous LDLR	*LDLR*	c.1871_1873del	Null/Null
39	F	Inclisiran	Homozygous LDLR	*LDLR*	c.97C>T	Null/Null
30	M	Placebo	Homozygous LDLR	*LDLR*	c.1729T>C	Non-Null/Null
38	M	Inclisiran	Homozygous LDLR	*LDLR*	c.1678A>T	Non-Null/Null
37	F	Placebo	Homozygous LDLR	*LDLR*	c.1690A>G (plus a single allele APOB c.3427C>T)	Non-Null/Null
70	F	Inclisiran	Homozygous LDLR	*LDLR*	c.858C>A	Non-Null/Null
48	F	Inclisiran	Homozygous LDLR	*LDLR*	c.2483A>G	Null/Null
32	F	Placebo	Homozygous LDLR	*LDLR*	c.2483A>G	Null/Null
35	F	Placebo	Homozygous LDLR	*LDLR*	c.941-?2140+?del	Null/Null
28	M	Inclisiran	Homozygous LDLR	*LDLR*	c.2483A>G	Null/Null
49	F	Placebo	Homozygous LDLR	*LDLR*	c.2483A>G	Null/Null
44	M	Inclisiran	Homozygous LDLR	*LDLR*	c.2483A>G	Null/Null
31	F	Inclisiran	Homozygous LDLR	*LDLR*	c.2483A>G	Null/Null

Age	Sex	Treatment	Genotype	Gene affected	Mutation type	LDLR function
22	F	Inclisiran	Homozygous LDLRAP1	*LDLRAP1*	c.345-2A>G	Non-Null/Null
27	F	Placebo	Homozygous LDLRAP1	*LDLRAP1*	c.345-2A>G	Non-Null/Null
31	M	Inclisiran	Homozygous APOB	*APOB*	c.10579C>T	Non-Null/Null
34	F	Inclisiran	Compound heterozygous LDLR	*LDLR*	c.530C>T; c.1054T>C	Non-Null/Null
41	F	Inclisiran	Compound heterozygous LDLR	*LDLR*	c.986G>A; c.1775G>A	Non-Null/Null
35	F	Placebo	Compound heterozygous LDLR	*LDLR*	c.1246C>T; c.940+3_940+6del	Non-Null/Null
29	F	Inclisiran	Compound heterozygous LDLR	*LDLR*	330del; c.1327T>C	Non-Null/Null
41	F	Inclisiran	Compound heterozygous LDLR	*LDLR*	c.681C>G; c.1285G>A	Null/Null
45	M	Placebo	Compound heterozygous LDLR	*LDLR*	c.268G>T; c.1951G>A	Non-Null/Null
26	M	Inclisiran	Compound heterozygous LDLR	*LDLR*	c.564C>G; Gain on Chr19:11230657-11234130	Null/Null
34	M	Inclisiran	Compound heterozygous LDLR	*LDLR*	c.268G>A; c.1729T>C	Non-Null/Null
42	F	Inclisiran	Compound heterozygous LDLR	*LDLR*	c.1567G>A; c.1988-50_2007del70	Non-Null/Null
56	M	Inclisiran	Compound heterozygous LDLR	*LDLR*	c.622G>A; c.858C>A	Non-Null/Null
50	F	Inclisiran	Compound heterozygous LDLR	*LDLR*	c.81C>G; c.590G>A	Non-Null/Null
58	M	Placebo	Compound heterozygous LDLR	*LDLR*	c.858C>A; c.1690A>G	Non-Null/Null

Age	Sex	Treatment	Genotype	Gene affected	Mutation type	LDLR function
62	M	Inclisiran	Compound heterozygous LDLR	*LDLR*	c.769C>T; c.1765G>A	Non-Null/Null
28	M	Inclisiran	Compound heterozygous LDLR	*LDLR*	c.986G>A; c.1747C>T	Non-Null/Null
23	M	Placebo	Compound heterozygous LDLR	*LDLR*	c.682G>A; c.1747C>T	Non-Null/Null
68	F	Inclisiran	Double heterozygous (LDLR+APOB)	*LDLR*; *APOB*	c.1567G>A; c.10579C>T	Non-Null/Null
66	M	Inclisiran	Double heterozygous (LDLR+APOB)	LDLR; *APOB*	c.268G>A; c.10579C>T	Non-Null/Null
48	F	Inclisiran	Other heterozygous	*LDLR*	c.1329G>A	Non-Null/Null
44	F	Inclisiran	Other heterozygous	*APOB*	c.10580G>A	Non-Null/Null
25	F	Placebo	Other heterozygous	*LDLR*	c.2416dupG	Non-Null/Null
47	F	Inclisiran	Other heterozygous	*LDLR*	c.1048C>T	Non-Null/Null
63	F	Inclisiran	Other heterozygous	*LDLR*	c.444T>G	Non-Null/Null
41	F	Placebo	Other heterozygous	*LDLR*	c.1322T>A	Non-Null/Null
64	F	Inclisiran	Other heterozygous	*LDLR*	c.501C>A	Non-Null/Null
62	F	Placebo	Other heterozygous	*LDLR*	c.1222G>A	Non-Null/Null
55	F	Placebo	Other heterozygous	*LDLR*	c.1186G>A	Non-Null/Null
32	M	Inclisiran	Other heterozygous	*LDLR*	c.1705+1G>A	Non-Null/Null
44	M	Placebo	Other heterozygous	*LDLR*	c.2416dupG	Non-Null/Null
56	M	Inclisiran	Other heterozygous	*APOB*	c.3337G>C	Non-Null/Null
34	F	Inclisiran	None identified	-	-	Non-Null/Null
50	M	Inclisiran	None identified	-	-	Non-Null/Null

Age	Sex	Treatment	Genotype	Gene affected	Mutation type	LDLR function
59	M	Placebo	None identified	-	-	Non-Null/Null
49	F	Inclisiran	None identified	-	-	Non-Null/Null
56	F	Inclisiran	None identified	-	-	Non-Null/Null

The data represents all randomized patients
APOB, apolipoprotein B; F, female; *LDLR*, low-density lipoprotein receptor; *LDLRAP1*, low-density lipoprotein receptor adaptor protein 1; M, male

Figures

Figure S1: Study design

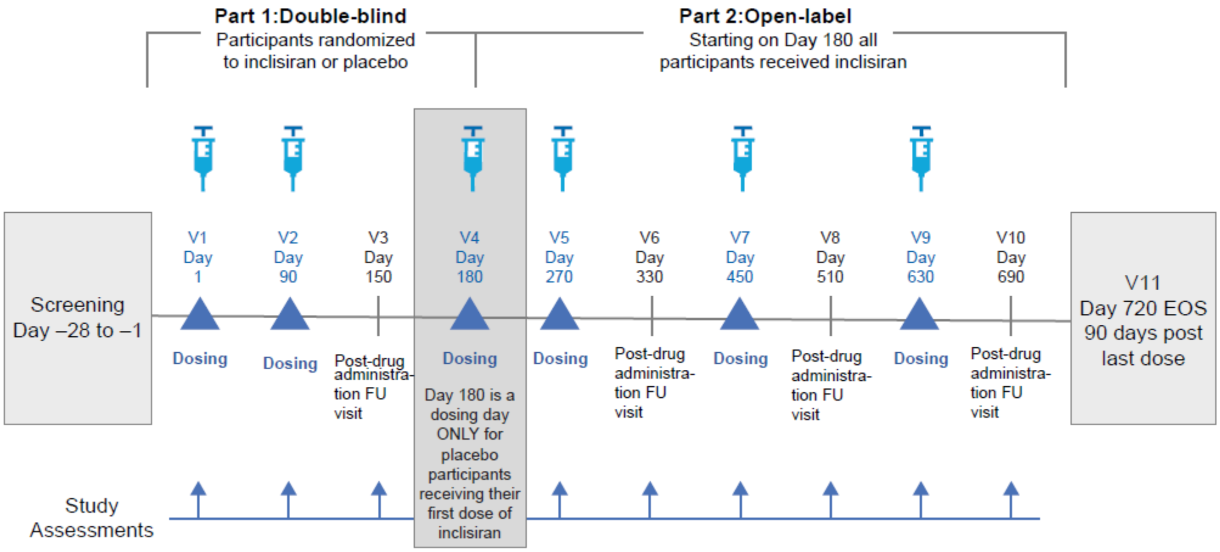

Part 1: Randomized 2:1 subcutaneous inclisiran sodium 300 mg versus placebo (1–6 months)
Part 2: Open-label subcutaneous inclisiran sodium 300 mg (6–24 months)
EOS, end of study; FU, follow-up; V, visit.

Figure S2: Patient disposition (ITT population)

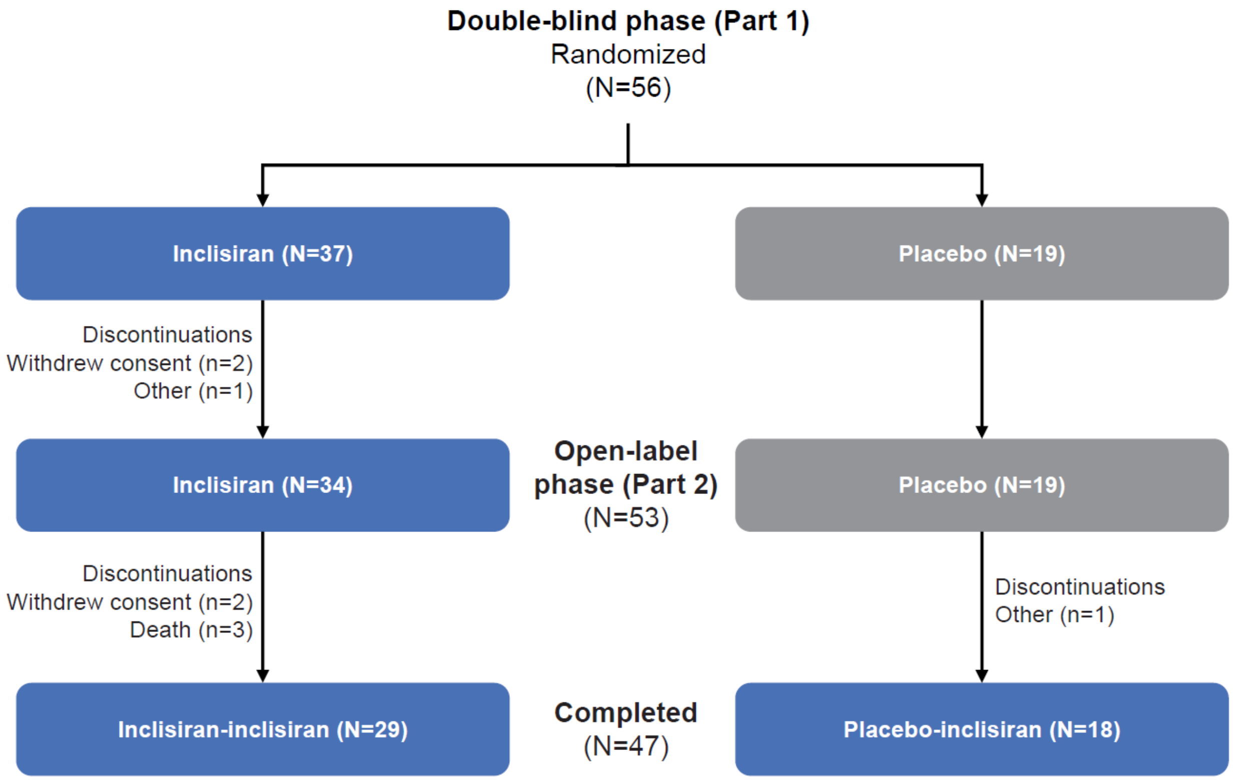

Part 1: Double blind, randomized 2:1 inclisiran sodium 300 mg versus placebo with maximally tolerated statins (1–6 months)
Part 2: Open-label inclisiran sodium 300 mg (6–24 months)
ITT, intent-to-treat; n, number of patients.

Figure S3: Mean observed percentage change from baseline in PCSK9 by visit and treatment (Part 1; ITT population)

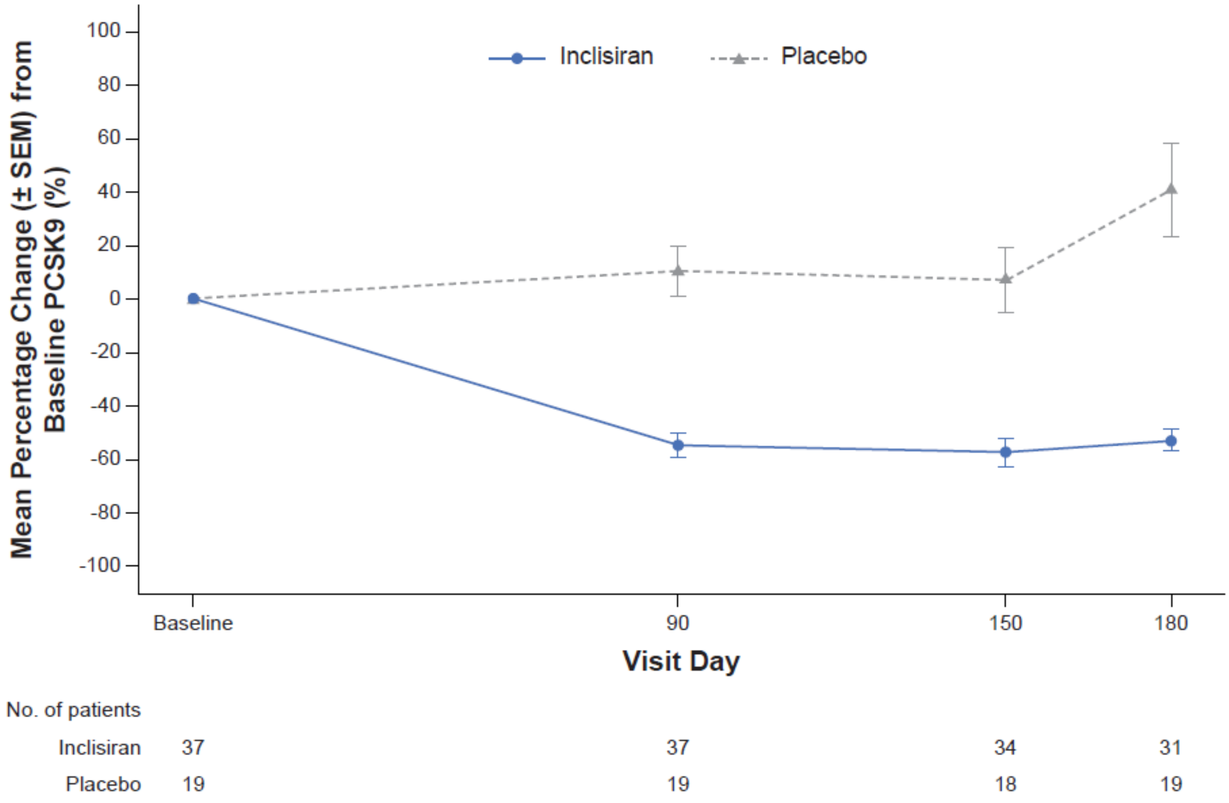

ITT, intent-to-treat; PCSK9, proprotein convertase subtilisin/kexin type 9; SEM, standard error of mean.

Figure Legends

Figure S1: Study design

Figure S2: Patient disposition (ITT population)

Figure S3: Mean observed percentage change from baseline in PCSK9 by visit and treatment (Part 1; ITT population)
